# The comparison of three different molecular imaging methods in localization and grading of insulinoma

**DOI:** 10.3389/fendo.2023.1163176

**Published:** 2023-06-30

**Authors:** Lina Chang, Xinyu Bi, Shuo Li, Qi Tong, Yian Gu, Zonghao He, Yansheng Li, Qiusong Chen, Jingqiu Cui, Haonan Yu, Qing He, Ming Liu

**Affiliations:** ^1^ Department of Endocrinology and Metabolism, Tianjin Medical University General Hospital, Tianjin, China; ^2^ Department of General Internal Medicine, Baodi District People’s Hospital, Tianjin, China; ^3^ Department of PET/CT Examination Room, Tianjin Medical University General Hospital, Tianjin, China

**Keywords:** grade, insulinoma, Ki-67, maximum standardized uptake value, molecular imaging, positron emission tomography/computed tomography (PET/CT)

## Abstract

**Aims:**

This cross-sectional study compared the value of molecular imaging (Exendin-4 positron emission tomography/computed tomography [PET/CT], ^68^Ga-DOTATATE PET/CT, ^18^F- fluorodeoxyglucose [FDG] PET/CT) in insulinoma localization by stratified tumor size and grading, and explored the correlation of the related the maximum standardized uptake value (SUVmax) with insulinoma grading, Ki-67, maximum tumor diameter, and glucose metabolism.

**Methods:**

In 28 insulinoma patients, the sensitivity of three types of PET/CT for localizing insulinoma was calculated according to tumor size and grade. We compared the SUVmax for different insulinoma grades and analyzed the correlation of SUVmax with Ki-67, maximum tumor diameter, and glucose metabolism indicators.

**Results:**

The study included 12 grade (G) 1 and 16 G2 cases, with maximum tumor diameters ranging from 9 to 40 mm. Without differentiation by size and grade, the sensitivity of Exendin-4 PET/CT to localize insulinoma was 100%, which significantly exceeded that of ^68^Ga-DOTATATE PET/CT and ^18^F-FDG PET/CT (75% and 57%, respectively). In tumors with a maximum diameter ≤ 20 mm and ≤ 15 mm, the sensitivity of Exendin-4 (both 100%) significantly exceeded that of ^68^Ga-DOTATATE PET/CT (74% and 64%, respectively) and ^18^F-FDG PET/CT (54% and 50%, respectively). In G1 tumors, the sensitivity of Exendin-4 PET/CT was significantly higher than that of ^18^F-FDG PET/CT, but not that of ^68^Ga-DOTATATE PET/CT, while in G2 tumors, the sensitivity of Exendin-4 PET/CT was significantly higher than that of both other types. However, all three PET/CT types missed a metastatic lymph node in one patient. The ^18^F-FDG PET/CT SUVmax was significantly lower than that of the other PET/CT types and that of ^68^Ga-DOTATATE PET/CT was significantly lower in G2 than in G1. ^68^Ga-DOTATATE PET/CT SUVmax correlated negatively with Ki-67. A receiver operating characteristic (ROC) curve suggested that ^68^Ga-DOTATATE PET/CT SUVmax > 19.9 could predict G1 tumors.

**Conclusion:**

Exendin-4 PET/CT was superior to ^68^Ga-DOTATATE PET/CT and ^18^F-FDG PET/CT for insulinoma localization, particularly small and G2 tumors, but its diagnostic value in small metastatic lymph nodes requires further exploration. ^68^Ga-DOTATATE PET/CT SUVmax could be used as an adjunct to pathology, and a value > 19.9 could predict G1 tumors. No PET/CT SUVmax could predict tumor maximum diameter and glucose metabolism.

## 1 Introduction

Neuroendocrine tumors (NETs) are rare tumors originating from neuroendocrine cells distributed throughout the body, of which gastrointestinal pancreatic NETs (GEP-NETs) is the most common, accounting for about two-thirds of all NETs (1). In the Chinese population, the pancreas is the most common site of GEP-NETs, and insulinoma is the most common functional pancreatic NET (PNET) (2, 3). Radical surgery is the first choice for treatment for insulinoma; thus, accurately locating the tumor before surgery is important. However, insulinomas are difficult to locate due to their small size (4, 5). Previous studies have found that the sensitivity of ^68^Ga-Exendin-4 positron emission tomography/computed tomography (PET/CT) targeting glucagon-like peptide-1 receptor (GLP-1R) to localize insulinoma could reach 97.7% (6). When ^68^Ga-Exendin-4 PET/CT is negative, ^68^Ga-DOTATATE PET/CT is recommended (7). In addition, ^18^F-fluorodeoxyglucose (FDG)-labeled PET/CT has certain diagnostic value for aggressive NETs (8).

To the best of our knowledge, no previous study has used all three types of PET/CT examinations for so many patients with insulinoma concurrently as in this study, or have compared the three types of PET/CT based on tumor size and tumor grade. Moreover, the maximum standardized uptake value (SUVmax) of ^68^Ga-DOTATATE PET/CT and ^18^F-FDG PET/CT have been shown to be correlated with Ki-67 in NETs (9–11). However, there has been no similar study on insulinoma in particular.

Here, we compared the three types of PET/CT based on tumor size and tumor grade, and analyzed the correlation of the SUVmax of the three types of PET/CT with Ki-67, maximum diameter of the tumor, and glucose metabolism in insulinoma patients.

## 2 Materials and methods

### 2.1 Study subjects

This retrospective study evaluated patients with endogenous hyperinsulinemic hypoglycemia who were highly suspected of having insulinoma who were hospitalized in the Department of Endocrinology and Metabolism of the Tianjin Medical University General Hospital, from September 2018 to March 2023. We included patients with insulinoma diagnosed by pathology; who underwent Exendin-4 PET/CT, ^18^F-FDG PET/CT, and/or ^68^Ga-DOTATATE PET/CT before surgery; who did not use hypoglycemic drugs; who had a negative insulin antibody screening result; who were not malnourished, pregnant, or lactating; and who had no other major diseases. We identified 28 patients who met these requirements.

This study received ethical approval from the institutional review board of Tianjin Medical University General Hospital (approval number: IRB2020-YX-029-01). The requirement for obtaining informed patient consent was waived because patients’ all information was extracted from electronic medical records, and patients’ identities were kept anonymous.

### 2.2 Anthropometric measurement and blood examination

The participants’ weight was measured using a calibrated scale, and their height was measured using a stadiometer. The body mass index (BMI) was calculated as the weight in kilograms divided by the height in square meters. Plasma glucose levels were measured using the hexokinase method. Serum insulin and C-peptide levels were measured using a chemiluminescent immunoassay.

### 2.3 Image acquisition and interpretation

Three types of PET/CT examinations were performed on different days by the machine of GE discovery 710. Tracers were injected through the dorsal vein of the hand. After resting quietly for 1 hour, Exendin-4/DOTATATE/FDG imaging and low-dose spiral CT tomography were performed. The imaging range was from the skull base to above the pubic symphysis. PET images were reconstructed by attenuation iteration to obtain sectional images, while CT sectional images were fused for multiple imaging. The images were interpreted in consensus by two experienced nuclear medicine physicians. ^18^F-FDG PET/CT was performed after 6 h of fasting, and no patient used somatostatin analogs before ^68^Ga-DOTATATE scanning. There was no fixed sequence for patients to undergo these three types of PET/CT examinations, and with a minimum interval of 24 hours between each two types of PET/CT. Positive lesions were generally defined as tracer concentrations that were nodular and above organ background levels. The SUVmax were measured for primary and metastatic lesions, and the highest SUVmax generated from each patient was used in the final analysis. Final PET/CT positive lesion was defined when any lesion in PET/CT was consistent with that in surgery, and the pathological results. For patients with negative ^18^F-FDG PET/CT or/and ^68^Ga-DOTATATE PET/CT, the SUVmax of the tumor location indicated by pathology was measured and was include into the final analysis.

### 2.4 Ki-67 and tumor grade

Ki-67 proliferation index values were measured on formalin fixed paraffin embedded tissue slices and Ki-67 was determined by calculating the average of 5 hot spot areas. A total of 500 cells in each spot were counted and results were expressed in percentage. Grade for patients with insulinoma was assigned based on the histological grading system used in the 2019 World Health Organization Classification. Three grades were defined as follows: G1 (Ki-67 < 3%), G2 (Ki-67 3–20%), and G3 (Ki-67 > 20%).

### 2.5 Statistical analysis

Continuous variables are shown as mean ± SD (normal distribution) or as median with interquartile ranges (skewed distribution), while categorical variables were expressed as numbers with percentages. Sensitivities and SUVmax values in different groups were compared by the chi-squared test and Mann–Whitney U tests. Spearman’s correlation coefficient was used to evaluate the correlation between SUVmax and Ki-67, maximum tumor diameter, and glucose metabolism indicators. ROC curve analysis was used to identify the cut-off value of ^68^Ga-DOTATATE PET/CT SUVmax to diagnose G1 insulinoma. Statistical analyses were performed using SPSS (version 23.0; IBM SPSS Inc., Armonk, NY, USA) and p < 0.05 was considered as statistically significant. The areas under the curve of blood glucose, insulin, and C-peptide in delayed oral glucose tolerance were calculated using GraphPad Prism 8.0.2 software. All figures in the article were drawn by GraphPad Prism 8.0.2 software.

## 3 Results

### 3.1 Clinical characteristics of the study subjects

Twenty-eight patients (11 males and 17 females) were included in this study. Blood glucose level was monitored in all patients during hospitalization, and 20 patients did 5-hour delayed oral glucose tolerance test. The nature of the tumor in all patients was confirmed as insulinoma by pathology, and two patients were finally diagnosed with multiple endocrine neoplasia(MEN) syndromes. There were 12 patients with grade (G)1 and 16 patients with G2 tumors, based on the World Health Organization classification in 2019. Twenty-seven patients had primary tumors in the pancreas and one patient had a primary tumor in the gastric antrum. The maximum diameters of the tumors ranged between 9 and 40 mm. Two patients had peripancreatic lymph node metastasis. The patients’ age, course of disease, lowest blood glucose level during hospitalization, insulin and C-peptide levels corresponding to the lowest blood glucose level, insulin release index (IRI), and area under the glucose (AUCglu), insulin (AUCins), C-peptide (AUCc-p) curve in the 5-hour delayed oral glucose tolerance test are shown in **Table 1**. All patients completed Exendin-4 PET/CT (2 patients were labeled with ^18^F, 26 patients were labeled with ^68^Ga) and ^18^F-FDG PET/CT, and 20 patients completed ^68^Ga-DOTATATE PET/CT.

**Table 1:** Characteristics of patients with insulinoma.

Patients (n = 28)	
Sex, n (%)	Male, 11 (39.29%)
	Female, 17 (60.71%)
Age (years)	54.68 ± 15.89
Body mass index (kg/m^2^)	25.85 ± 4.08
Duration of hypoglycemia (months)	24.00 (12.00–60.00)
Lowest blood glucose level (mmol/L)	1.87 ± 0.56
Insulin when lowest blood glucose level (mU/L)	15.10 (12.10–28.40)
C–peptide when lowest blood glucose level(ng/ml)	3.11 (2.38–4.75)
IRI	0.45 (0.35–1.13)
AUCglu	27.14 ± 6.89
AUCins	288.48 ± 149.64
AUCc–p	33.65 ± 13.15
Grade, n (%)	G1, 12/28 (42.86%)
	G2, 16/28 (57.14%)
Ki–67 (%)	3.00 (2.00–4.00)
Maximum tumor diameter (mm)	15.00 (12.00–20.00)

IRI, insulin release index = insulin (mU/L)/(glucose (mmol/L) × 18).

AUC, area under the curve.

### 3.2 Sensitivity of three PET/CT imaging modalities

**Figure 1:**
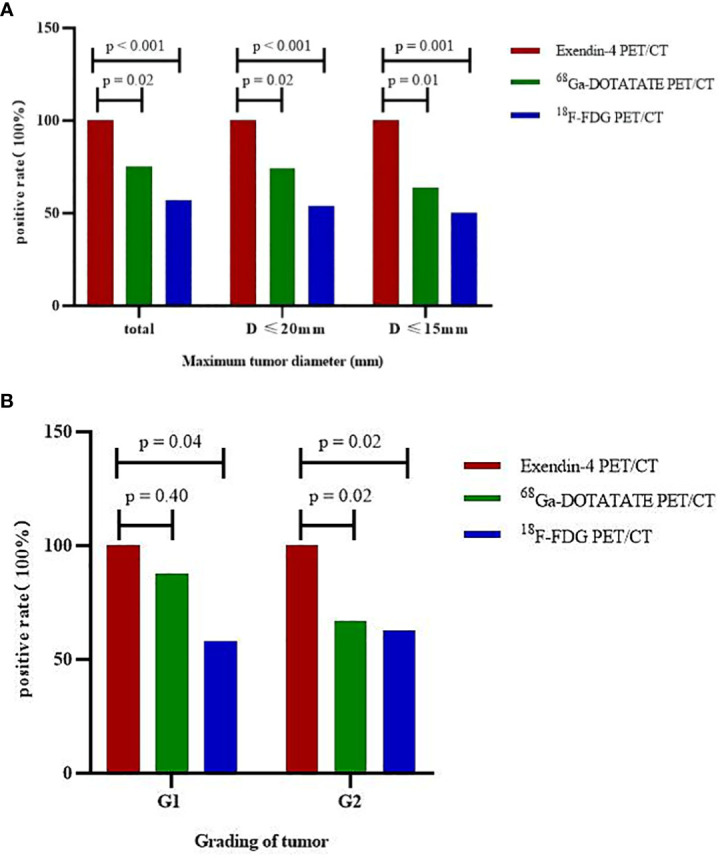
Sensitivity of three PET/CT imaging modalities to locate insulinoma by stratified tumor size and grading. **(A)** tumors of different sizes, **(B)** tumors of different grades.

When analyzing all tumors together, the sensitivity of Exendin-4 PET/CT for locating insulinoma was 100%, which was significantly higher than that of ^68^Ga -DOTATATE PET/CT (p = 0.02, 95% confidence interval (95% CI) 1.04-1.72)and ^18^F-FDG PET/CT (p < 0.001, 95% CI 1.27-2.41). In tumors with a maximum diameter ≤ 20 mm and ≤ 15 mm, the sensitivity of Exendin-4 PET/CT was 100% for both, which was still significantly higher than that of ^68^Ga-DOTATATE PET/CT (p = 0.02, 95% CI 1.04-1.78; p = 0.01, 95% CI 1.05-2.30) and ^18^F-FDG PET/CT (p < 0.001, 95% CI 1.30-2.65; p = 0.001, 95% CI 1.26-3.17). The sensitivity advantage of ^68^Ga-Exendin-4 PET/CT was more obvious as the tumor diameter reduced. These results were shown in **Table 2** and **Figure 1A**.

**Table 2:** Three PET/CT positive rates in stratified analysis according to the tumor size.

	Total	Positive rate	p	Dmax ≤ 2 cm	Positive rate	P	Dmax ≤ 1.5 cm	Positive rate	p
Exendin-4 PET/CT	28	1.00	NA	26	1.00	NA	18	1.00	NA
^68^Ga-DOTATATE PET/CT	20	0.75	0.02^a^	19	0.74	0.02^a^	14	0.64	0.01^a^
^18^F-FDG PET/CT	28	0.57	< 0.001^a^	26	0.54	< 0.001^a^	18	0.50	0.001^a^

a: Compared with Exendin-4 PET/CT, p < 0.05; NA, not available.

For G1 patients (n = 12), the sensitivity of Exendin-4 PET/CT to localize insulinoma was 100%, which was significantly higher than that of ^18^F-FDG PET/CT (p = 0.04, 95% CI 1.06-2.77) but not that of ^68^Ga-DOTATATE PET/CT. For G2 (n = 16) patients, the sensitivity of Exendin-4 PET/CT to locate insulinoma was 100%, which was significantly higher than that of ^68^Ga-DOTATATE PET/CT (p = 0.02, 95% CI 1.01-2.24) and ^18^F-FDG PET/CT (p = 0.02, 95% CI 1.10-2.34). These results are shown in **Table 3** and **Figure 1B**. However, there was no significant difference in the sensitivity of ^68^Ga-DOTATATE PET/CT and ^18^F-FDG PET/CT in the localization of insulinoma when the tumor size and grading were analyzed hierarchically.

**Table 3:** Three PET/CT positive rates in stratified analysis according to the grading of tumor.

	G1			G2		
	Number	Positive rate	p	Number	Positive rate	p
Exendin-4 PET/CT	12	1.00	NA	16	1.00	NA
^68^Ga-DOTATATE PET-CT	8	0.88	0.40	12	0.67	0.02^a^
^18^F-FDG PET-CT	12	0.58	0.04^a^	16	0.63	0.02^a^

a: Compared with Exendin-4 PET/CT, p < 0.05; NA, not available.

In the above analysis, any lesion found in a patient was considered positive. However, if all positive lesions were used as the positive standard, the sensitivity of Exendin-4 PET/CT to locate tumor was not 100%, because it missed a metastatic lymph node in one patient. The pathological characteristics of this patient were as follows, the primary tumor was located in the tail of the pancreas, with a diameter of 26 mm, grading was G2, and Ki-67 was 3%, and the maximum diameter of the metastatic lymph node in the tail of the pancreas was 5.5 mm, which was missed by all three PET/CT imaging modalities. However, Exendin-4 PET/CT successfully located the metastatic lymph node(with a maximum diameter of 12.4 mm) of another patient. The metastatic lymph node of the former patient may not have been located due to the small size of the lymph node or because it did not express GLP-1R.

### 3.3 SUVmax among different groups

**Figure 2:**
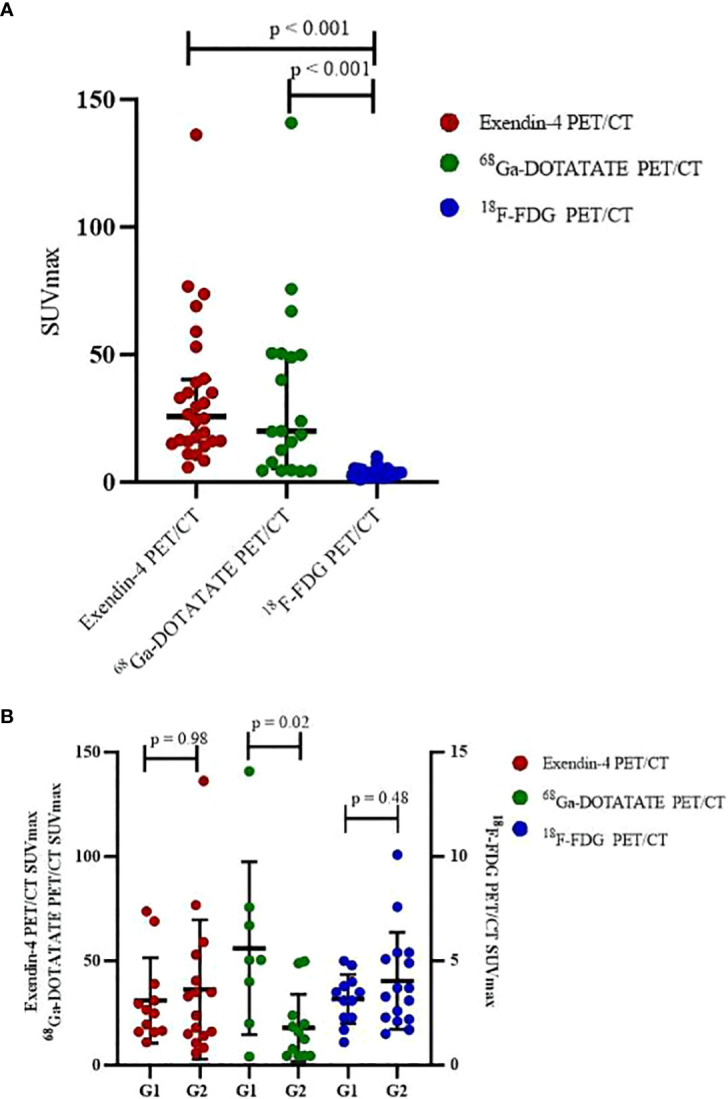
Comparison SUVmax of three PET/CT **(A)** total, **(B)** in different grades.

Comparing SUVmax of the three PET/CT revealed that both SUVmax of Exendin-4 PET/CT (p < 0.001, 95% CI of median difference 14.20-30.40) and ^68^Ga-DOTATATE PET/CT (p < 0.001, 95% CI of median difference 9.50-43.50) were higher significantly than that of ^18^F-FDG PET/CT (**Table 4**, **Figure 2A**). Comparing SUVmax of G1 and G2 tumors revealed that the ^68^Ga-DOTATATE PET/CT SUVmax was significantly higher in G1 than in G2 tumors (p = 0.02, 95% CI of median difference 1.60-60.00), while Exendin-4 PET/CT SUVmax and ^18^F-FDG PET/CT SUVmax did not differ statistically significantly between the two grades (**Table 4**, **Figure 2B**).

**Table 4:** Comparison of three PET-CT SUVmax.

	total	G1	G2
Exendin-4 PET/CT SUVmax	30.30 (16.05-54.38)	30.30(18.23-61.53)	29.50(15.38-54.38)
^68^Ga-DOTATATE PET-CT SUVmax	19.90 (5.48-50.28)	50.45(25.00-73.60)^c^	14.20(4.63-22.88)
^18^F-FDG PET-CT SUVmax	3.20 (2.23-4.68)^ab^	3.30(2.30-3.95)	3.20(2.13-5.28)

a: compare with Exendin-4 PET/CT, p<0.05; b: compare with ^68^Ga-DOTATATE PET/CT, p<0.05; c: compare with G2, p<0.05.

### 3.4 Correlation between SUVmax and Ki-67

**Figure 3:**
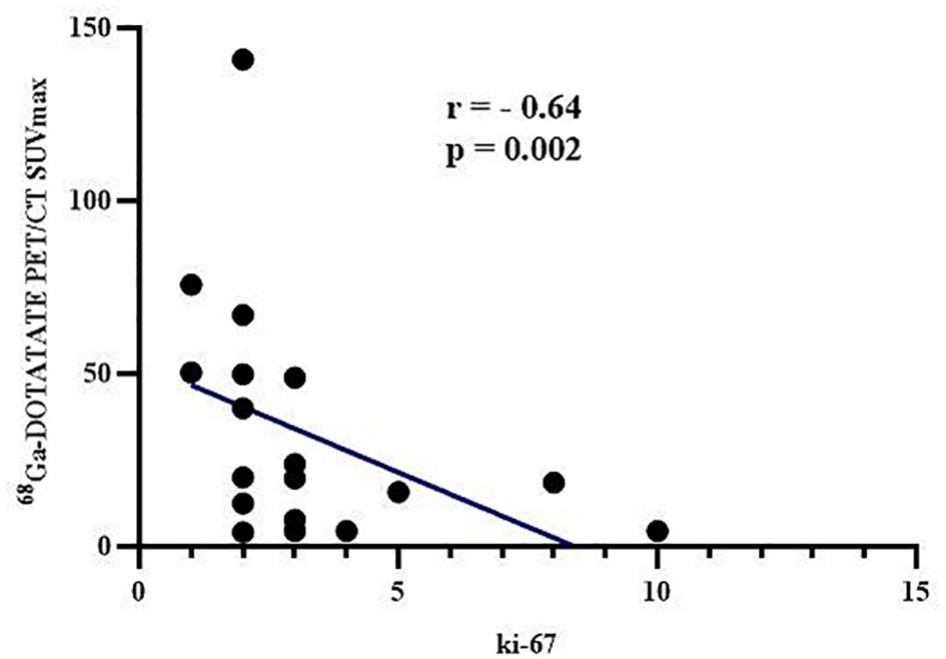
Correlation between ^68^Ga-DOTATATE PET/CT SUVmax and Ki-67.

The ^68^Ga-DOTATATE PET/CT SUVmax correlated negatively with tumor Ki-67 (r = - 0.64, p = 0.002, **Figure 3**), which was consistent with the result that ^68^Ga-DOTATATE PET/CT SUVmax in patients with G1 was higher than that of patients with G2 tumors (**Table 4**). However, Exendin-4 PET/CT SUVmax and ^18^F-FDG PET/CT SUVmax showed no correlation with Ki-67 (**Table 5**). In addition, there were no correlations of the three PET/CT SUVmax values with the maximum tumor diameter, the lowest blood glucose level and corresponding insulin and C-peptide level, IRI, AUCglu, AUCins, and AUCc-p (**Table 5**).

**Table 5:** Correlations of SUVmax with tumor maximum diameter and glucose metabolism.

	Exendin-4 PET/CT SUVmax	^68^Ga-DOATATE PET/CT SUVmax	^18^F-FDG PET/CT SUVmax
Ki–67 (%)	r = 0.04, p = 0.84	r = - 0.64, p = 0.002^*^	r = 0.29, p = 0.14
Maximum tumor diameter (mm)	r = - 0.05, p = 0.80	r = - 0.02, p = 0.93	r = 0.32, p = 0.10
Lowest blood glucose level(mmol/L)	r = 0.15, p = 0.44	r = 0.38, p = 0.10	r = - 0.06, p = 0.77
Insulin when lowest blood Glucose level (mU/L)	r = - 0.07, p = 0.71	r = 0.27, p = 0.24	r = 0.16, p = 0.42
C–peptide when lowest blood glucose level(ng/ml)	r = - 0.21, p = 0.29	r = 0.12, p = 0.64	r = 0.19, p = 0.34
IRI	r = - 0.003, p = 0.99	r = 0.23, p = 0.34	r = 0.07, p = 0.71
AUCglu	r = - 0.19, p = 0.42	r = - 0.21, p = 0.43	r = -0.30, p = 0.21
AUCins	r = - 0.05, p = 0.83	r = 0.20, p = 0.46	r = 0.40, p = 0.09
AUCc–p	r = - 0.08, p = 0.75	r = 0.14, p = 0.59	r = 0.27, p = 0.24

IRI, insulin release index = insulin (mU/L)/(glucose (mmol/L) × 18); AUC, area under the curve.

**Figure 4:**
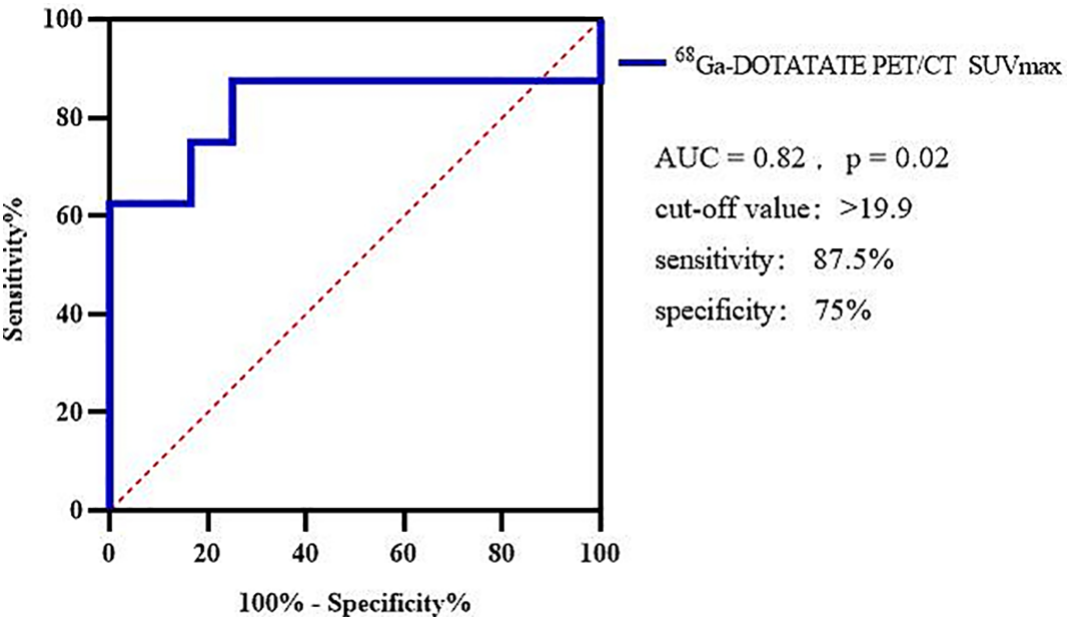
Using ^68^Ga-DOTATATE PET/CT SUVmax to differentiate G1 and G2.

Using ^68^Ga-DOTATATE SUVmax to differentiate between G1 and G2, the AUC of the ROC curve was 0.82 and the cutoff point of SUVmax was 19.9, sensitivity was 87.5%, and specificity was 75% (**Figure 4**).

## 4 Discussion

At present, surgical treatment remains the first choice for patients with insulinoma, and it is the only method by which cure can be achieved. Thus, accurate preoperative localization is particularly important. However, most insulinomas are small, with 82% being smaller than 2 cm in diameter, and 47% being smaller than 1 cm in diameter (5). Therefore, it is difficult to locate these tumors accurately before operation. Preoperative localization examinations include abdominal plain CT and magnetic resonance imaging (MRI), endoscopic ultrasound (EUS), arterial calcium-stimulated hepatic vein blood sampling (ASVS), molecular imaging (such as ^18^F-FDG PET/CT, Exendin-4 PET/CT, somatostatin receptor(SSTR) imaging).

### 4.1 Disadvantages of conventional imaging

For pancreatic neuroendocrine tumors with diameter < 1.0 cm, the detection rate of abdominal plain CT and MRI is < 15%. Even if the tumor diameter exceeds 1.0 cm, the detection rate is only 60–90% (12). Studies have shown that the sensitivity and specificity of multi-slice spiral biphasic CT combined with pancreatic perfusion scanning for localization of insulinoma can be as high as 94.6% and 94.7%, respectively (13). However, about one-fourth of insulinomas show equal density enhancement at each enhancement stage, and one-third of insulinomas show transient enhancement, which can easily be missed (14, 15). During EUS examination, the ultrasonic probe is closer to the pancreas; thus, it is particularly suitable for identifying small pancreatic lesions, and can detect lesions as small as 5 mm. However, the sensitivity of EUS for detecting insulinoma in the head of the pancreas is higher than that for detecting insulinoma in the tail of pancreas. Consequently, the insulinoma detection rate of EUS varies from 40% to 92.6%, and EUS cannot be used to assess distant metastases (16–18). ASVS stimulates insulinoma to secrete insulin by selectively injecting calcium gluconate into the pancreatic artery to locate the tumor. This method is accurate, however, it is an invasive examination, and it cannot accurately display the location of a lesion when there are multiple foci of insulinoma, and it cannot locate ectopic insulinoma (19, 20).

### 4.2 Research status of molecular imaging

GLP-1R is a G protein-coupled receptor that regulates insulin secretion and is an important target for diagnosis of insulinoma (21). GLP-1R is the receptor with the highest expression level on the surface of insulinoma cells, which is about 6–12 times that of normal β cells, and GLP-1R is only expressed to a low degree or not expressed in other pancreatic tumors or non-tumor lesions (22, 23). Jeans et al. studied the expression of 13 peptide receptors on the surface of insulinoma cells and found that GLP-1R, cholecystokinin-2, and vasoactive intestinal peptide-1 receptors had the highest incidence in insulinoma, but the density of the latter two was far lower than that of GLP-1R (24). Exendin-4 is a molecular tracer targeting GLP-1R; thus, it has high sensitivity and specificity in the diagnosis of insulinoma. It has been reported that the sensitivity of ^68^Ga-Exendin-4 PET/CT for the localization of insulinoma is as high as 97.7% (6). ^68^Ga-DOTATATE PET/CT was approved by the FDA for evaluation of patients with gastrointestinal pancreatic neuroendocrine neoplasms worldwide, as it could specifically bind to SSTRs that are overexpressed on the surface of their cells, particularly subtypes 2 and 5 (25). However, due to the low expression level of SSTR in insulinoma, the effect of somatostatin receptor imaging in the localization of insulinoma is poor, and the rate of missed diagnosis is high, so that it is not recommended as a first-line examination method (26). Another radiopharmaceutical used in PET imaging is FDG; however, insulinoma demonstrates little or no FDG uptake, due to their small size and low metabolic activity, whereas poorly differentiated NET are well depicted in ^18^F-FDG PET/CT (27, 28). No previous study has used all three types of PET/CT examinations for so many patients with insulinoma concurrently as in this study, or have compared the three types of PET/CT based on tumor size and tumor grade. Our data showed that the SUVmax of ^18^F-FDG PET/CT is significantly lower than that of the other two PET/CT modalities in G1 and G2 insulinoma. In addition, the localization value of Exendin-4 PET/CT for insulinoma is better than that of ^68^Ga-DOTATATE PET/CT and ^18^F-FDG PET/CT, which is consistent with previous reports. Through further hierarchical analysis, we found that Exendin-4 PET/CT had more obvious localization advantages in small tumors and in G2 tumors. However, Exendin-4 PET/CT missed the diagnosis of a metastatic lymph node in one patient. It was previously reported that ^68^Ga-Exendin-4 PET/CT missed insulinomas with a diameter of less than 1 cm with a rate of 1.9% (29). It was considered that the metastatic lymph node that was not accurately located in our study might be due to the small size of the lymph node or because it did not express GLP-1R.

Recently, it has been found that ^68^Ga-DOTATATE PET/CT SUVmax is negatively correlated with Ki-67, and ^18^F-FDG PET/CT SUVmax is positively correlated with Ki-67 in neuroendocrine tumors (10, 11). Another study found that ^68^Ga-DOTATATE PET/CT SUVmax and Ki-67 were negatively correlated in gastrointestinal tumors, while no such correlation was found in pancreatic neuroendocrine neoplasms (25). At present, there is no study on the three types of PET/CT SUVmax and Ki-67 or grade of insulinoma in particular. We found that ^68^Ga-DOTATATE PET/CT SUVmax in insulinoma patients with G2 was lower than G1, and ^68^Ga-DOTATATE PET/CT SUVmax correlated negatively with Ki-67 expression. Further ROC curve analysis found that the cut-off point of ^68^Ga-DOTATATE PET/CT SUVmax for distinguishing G1 and G2 insulinoma was 19.9. Ki-67 has guiding significance for prognosis, but limited tissue availability may hinder accurate evaluation of Ki-67 in some cases, which may lead to miscalculation of tumor grading. In addition, the Ki-67 index of the same patient may change with disease progression. As a non-invasive general examination method, ^68^Ga-DOTATATE PET/CT can supplement histopathology and may help overcome some limitations of Ki-67 assessments, including insufficient tissue sampling, tumor heterogeneity, technical problems related to histopathology, and variability in scoring among observers (11, 30).

Due to the different advantages of those three types of PET/CT in the diagnosis of insulinoma as mentioned above and to exclude hypoglycemia caused by nonislet cell tumours, we conducted three types of PET/CT examinations after acquiring patients’ informed consent. Due to the unstable sequence of these three types of PET/CT examinations, some patients refused to undergo further ^68^Ga-DOTATATE PET/CT examinations after positive results on Exindin-4 PET/CT or/and ^18^F-FDG PET/CT, so not all individuals underwent ^68^Ga-DOTATATE PET/CT examination in this study.

### 4.3 Novelties and limitations

The novelty of our article lies in the comparison of three types of PET/CT imaging for so many patients with insulinoma concurrently according to tumor size and tumor grade, and the analysis of correlation between SUVmax and Ki-67 in insulinoma in particular. The limitations of our study were that the sample size was relatively small and that there were no G3 or pancreatic neuroendocrine carcinoma patients. However, our results have deepened clinicians’ understanding of the relationship between molecular imaging and insulinoma and have certain implications for further research.

## 5 Conclusion

In conclusion, the Exendin-4 PET/CT showed superior insulinoma location ability as compared to ^68^Ga-DOTATATE PET/CT and ^18^F-FDG PET/CT, particularly for detecting small tumors and G2 tumors, but its diagnostic value in small metastatic lymph nodes requires further exploration. ^68^Ga-DOTATATE PET/CT SUVmax was negatively correlated with Ki-67; thus, it could be used to supplement a pathology-based diagnosis. This SUVmax > 19.9 could predict G1 tumors. The SUVmax of Exendin-4 PET/CT, ^68^Ga-DOTATATE PET/CT, and ^18^F-FDG PET/CT had no predictive value for tumor maximum diameter and glucose metabolism.

## Data availability statement

The original contributions presented in the study are included in the article/supplementary material. Further inquiries can be directed to the corresponding authors.

## Ethics statement

The studies involving human participants were reviewed and approved by Tianjin Medical University General Hospital (approval number: IRB2020-YX-029-01). Written informed consent for participation was not required for this study in accordance with the national legislation and the institutional requirements.

## Author contributions

Conceived and designed the study: ML, QH and HY. Performed the study: LC, XB and SL. Analyzed the data: LC, SL and QT. Provided imaging guidance:YL and QC. Collected cases information: YG, ZH and JC. Wrote the original draft of the manuscript: LC. Supervised this work: ML and QH. All authors contributed to the article and approved the submitted version.
